# Ethylene Supports Colonization of Plant Roots by the Mutualistic Fungus *Piriformospora indica*

**DOI:** 10.1371/journal.pone.0035502

**Published:** 2012-04-19

**Authors:** Behnam Khatabi, Alexandra Molitor, Christian Lindermayr, Stefanie Pfiffi, Jörg Durner, Diter von Wettstein, Karl-Heinz Kogel, Patrick Schäfer

**Affiliations:** 1 Research Centre for Biosystems, Land Use, and Nutrition, Institute of Phytopathology and Applied Zoology, Justus Liebig University, Giessen, Germany; 2 Helmholtz Zentrum München - German Research Center for Environmental Health Institute of Biochemical Plant Pathology, Oberschleissheim, Germany; 3 Department of Crop and Soil Sciences, Washington State University, Pullman, Washington, United States of America; 4 School of Molecular Biosciences, Washington State University, Pullman, Washington, United States of America; Texas A&M University, United States of America

## Abstract

The mutualistic basidiomycete *Piriformospora indica* colonizes roots of mono- and dicotyledonous plants, and thereby improves plant health and yield. Given the capability of *P. indica* to colonize a broad range of hosts, it must be anticipated that the fungus has evolved efficient strategies to overcome plant immunity and to establish a proper environment for nutrient acquisition and reproduction. Global gene expression studies in barley identified various ethylene synthesis and signaling components that were differentially regulated in *P. indica*-colonized roots. Based on these findings we examined the impact of ethylene in the symbiotic association. The data presented here suggest that *P. indica* induces ethylene synthesis in barley and *Arabidopsis* roots during colonization. Moreover, impaired ethylene signaling resulted in reduced root colonization, *Arabidopsis* mutants exhibiting constitutive ethylene signaling, -synthesis or ethylene-related defense were hyper-susceptible to *P. indica*. Our data suggest that ethylene signaling is required for symbiotic root colonization by *P. indica*.

## Introduction

Ethylene plays a prominent role in senescence and plant development [1], [2]. In *Arabidopsis thaliana*, ethylene is perceived by five ER membrane-bound receptors (e.g. Ethylene Triple Response 1, ETR1). In the absence of ethylene, the receptors activate a Raf-like kinase (Constitutive Triple Response 1, CTR1), which negatively regulates the downstream ethylene response pathway [3]. Binding of ethylene inactivates the receptors, resulting in the deactivation of CTR1, which allows downstream effectors such as Ethylene Insensitive 2 (EIN2) to function as positive regulators of ethylene signaling [4], [5] by activating transcription factors Ethylene Insensitive 3 (EIN3) and EIN3-like 1 (EIL1) [6]. Constitutive ethylene signaling is observed in *ctr1* [3] and in *ethylene overproducer 1* (*eto1*) mutants. ETO1 negatively regulates ethylene synthesis by inactivating and/or degrading 1-aminocyclopropane-1-carboxylic acid synthase 5 (ACS5) and probably other ACS isoforms such as ACS4, ACS8, and ACS9 [7], [8], [9]. It has long been known that ethylene supports plant immunity [2]. For instance, EIN3 and EIL1 drive the expression of primary ethylene transcriptional activators, such as *Ethylene Response Factor 1* (*ERF1*). ERF1 regulates ethylene responsive and defense-related genes (e.g. *Pathogenesis-related 3*, *Plant Defensin 1.2*) [10] thereby contributing to defense against necrotrophic pathogens [11]. Recent studies underlined the participation of ethylene in very early processes of immune signaling [12], [13]. Plant immunity is induced after perception of conserved microbial molecules, so called microbe-associated molecular patterns (MAMPs, e.g. flagellin, chitin), by specific pattern recognition receptors (PRRs) [14]. The recognition of bacterial flagellin by the PRR Flagellin Sensing 2 (FLS2) results in the activation of an array of immune responses summarized as MAMP-triggered immunity (MTI), and includes the rapid production of reactive oxygen species (ROS) as well as ethylene [15]. It has been shown that ethylene signaling is essential for flagellin-triggered ROS production [13], [16]. In a model proposed by Boutrot *et al.* (2010), flagellin recognition by FLS2 results in MAP kinase (MAPK) 3 and 6 phosphorylation that, in turn, phosphorylates and thereby stabilizes ACS2, ACS6, and EIN3 [12], [16], [17], [18]. Consequently, rapid ethylene production is immediately downstream of MAMP recognition, and, due to the transcriptional regulation of *FLS2* by EIN3, ethylene mediates a steady-state level of FLS2 at the plasma membrane [13], [16]. By contrast, impaired ethylene signaling disturbs FLS2 regulation, subsequent MAPK3/6 phosphorylation and ROS production, processes that are required to stop pathogen invasions. Thus, ethylene has a more complex role in the activation of early and late immune responses.

*Piriformospora indica* is a root-colonizing basidiomycete that colonizes mono- and dicotyledonous plants, including barley (*Hordeum vulgare*) and *Arabidopsis*, in which the fungus increases yield and adaptation to abiotic and biotic stress [19], [20], [21], [22], [23]. Cytological and genetic studies have shown that *P. indica* initially colonizes living cells. This biotrophic growth phase is observed up to 3 days after inoculation [24] and is followed by a second cell death-dependent colonization phase (>3 dai), which is restricted to colonized cells [24], [25], [26]. The fungus has an immune suppressing activity, which is essential for biotrophic root colonization, and may particularly explain its remarkably broad host range [24]. DNA microarray-based gene expression analysis of barley roots colonized by *P. indica* showed the differential expression of genes related to ethylene synthesis and signaling [27]. In the present study, we therefore analyzed the effect of ethylene on the colonization of *Arabidopsis* and barley roots by *P. indica*. We demonstrate that *P. indica* induces 1-aminocyclopropane-1-carboxylic acid (ACC) synthesis and that ethylene signaling is not detrimental to fungal growth. We discuss the possibility that ethylene is a positive modulator of the mutualistic plant root-*P. indica* symbiosis.

## Results

### Impaired ethylene signaling reduces colonization of plant roots by *P. indica*

Global transcriptome analyses revealed differential regulation of components with putative functions in ethylene synthesis and signaling in barley roots inoculated with *P. indica* [27]. Among the ethylene synthesis genes were three *1-aminocyclopropane-1-carboxylic acid (ACC) oxidases* (Table 1). Six genes involved in signaling encoded putative transcription factors: *ethylene-responsive element binding protein, ethylene insensitive 3-like 2, AP2 domain transcription factor EREBP,* a putative *RAV2-like DNA binding protein, ethylene-responsive factor*, and *ethylene-binding protein-like* (Table 1). Interestingly, while ethylene synthesis genes were mostly induced, signaling components were generally suppressed during *P. indica* colonization (Table 1).

**Table 1:** List of barley genes differentially regulated by *P. indica* and involved in ethylene synthesis or signaling.

		Fold change (dai)			
Gene^1^	Acc. number	1	3	7	Process
*1-aminocyclopropane-1-carboxylate oxidase*	ABM74187.1	−2,3	-	-	synthesis
*putative 1-aminocyclopropane-1-carboxylic acid oxidase*	BAB84460.1	-	4,3	-	synthesis
*putative 1-aminocyclopropane-1-carboxylate oxidase*	AAU44031.1	-	2,6	2,8	synthesis
*AP2 domain transcription factor EREBP*	AAP56251.1	-	2,0	-	signaling
*ethylene-binding protein-like*	BAD38371.1	-	-	−3,3	signaling
*ethylene insensitive 3-like 2*	AAV68140.1	−3,1	-	-	signaling
*ethylene-responsive element binding protein*	ABO93372.1	4,3	-	-	signaling
*ethylene-responsive factor*	ABQ52686.1	-	−2,6	-	signaling
*Similar to probable RAV2-like DNA binding protein*	AAX92718.1	-	−2,2	-	signaling

1 Gene expression data was published in [27].

**Figure 1:**
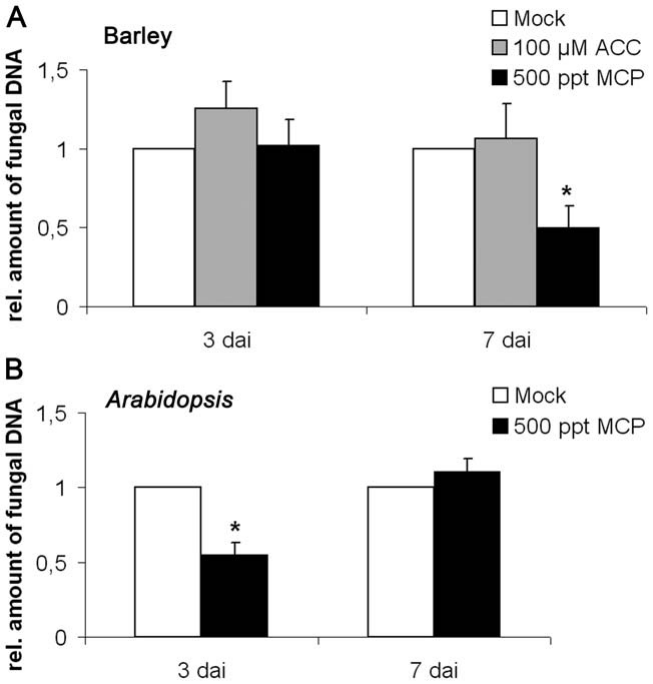
Colonization of barley and *Arabidopsis* by *P. indica* in response to ACC and MCP. (**A**) Two-day-old barley seedlings or (**B**) two-week-old *Arabidopsis* seedlings were inoculated with *P. indica* and subsequently treated with 500 ppt 1-methylcyclopropene (MCP) as described in Materials and Methods. Barley was also treated with 100 µM 1-aminocyclopropane-1-carboxylic acid (ACC). MCP inhibited *P. indica* colonization at 3 or 7 dai in *Arabidopsis* or barley, respectively. The values are normalized to colonization in mock-treated roots (set to one). The data are based on three independent biological experiments. Student's *t*-test indicates a significant difference in *P. indica*-colonization of MCP-treated roots (* P<0.05).

The data raised the possibility that ethylene modulates *P. indica*'s ability to colonize plant roots. Because barley mutants with compromised ethylene biosynthesis and signaling are not available, we conducted tentative pharmacological experiments in order to determine the significance of ethylene at early stages of a successful symbiosis. To this end, two-day-old barley seedlings were transferred to agar plates containing 100 µM of the ethylene precursor 1-aminocyclopropane-1-carboxylic acid (ACC), or to a jar containing a vial with 1 mM of the ethylene antagonist 1-methylcyclopropene (MCP), which blocks ethylene signaling by interacting with ethylene receptors [28]. Seedlings were inoculated with *P. indica* (500,000 chlamydospores ml^−1^) and fungal colonization was determined at 3 and 7 days after inoculation (dai) by quantitative real time PCR (qRT-PCR). While root colonization was unaltered after ACC treatment, MCP treatment resulted in an approximately 50% reduction in the amount of fungal DNA at 7 dai (Students *t*-test, P<0.05) (Figure 1A). Neither of the compounds had adverse effects on morphology or growth of *P. indica in vitro* (not shown). We further tested whether ethylene might generally affect colonization of different plant hosts. Upon treatment of *Arabidopsis* with MCP, *P. indica* also displayed reduced root colonization, although in contrast to barley, the effect was already detectable at 3 dai (Figure 1B). These data suggest that colonization of barley and *Arabidopsis* roots by *P*. *indica* is supported by ethylene signaling.

### ACC levels are increased in *P. indica*-colonized barley roots

**Figure 2:**
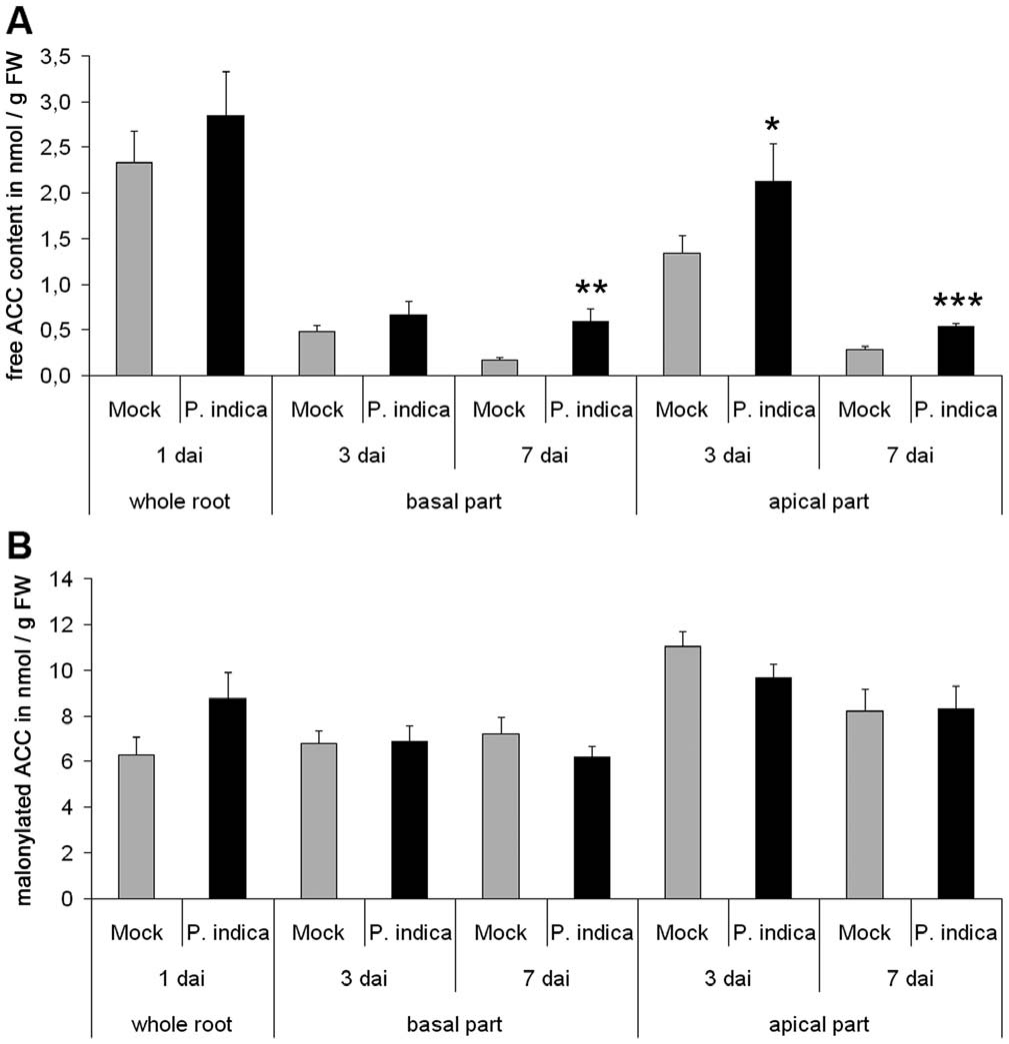
ACC content in barley roots during *P. indica* colonization. Free (**A**) and malonylated (**B**) 1-aminocyclopropane-1-carboxylic acid (ACC) contents were determined in *P. indica* and mock-treated roots at 1, 3, and 7 days after treatments. At 1 dai, the complete roots were harvested and forwarded to ACC measurements. At 3 and 7 dai, the upper two centimeters (basal part) and the remaining part of the roots (apical part) were analyzed separately. Absolute values are given in nmol • g FW^−1^ for mock-treated and *P. indica*-colonized roots. (**A**) Free ACC levels were significantly enhanced at 3 and 7 dai in the apical zone and 7 dai in the basal part as indicated by Students *t*-test (* P<0.05, ** P<0.01, *** P<0.001). (**B**) Malonylated ACC was not significantly altered during *P. indica* colonization at any timepoint or in any tissue. Data show the mean content of four biological experiments (with at least two technical repetitions per experiment) and bars indicate standard errors.

While blockage of ethylene signaling reduced fungal colonization of barley, application of ACC, the immediate precursor of ethylene, had no effect. One explanation could be that ACC levels were high per se in young roots regardless of fungal colonization. If ethylene signaling was indeed saturated, treatment with ACC would not further affect ethylene synthesis and thus fungal root colonization. To test this hypothesis, we determined ACC contents in P. indica-colonized roots. The ACC pool in plants constists of free and malonylated ACC. Malonylation is a mean to inactivate and thereby control the amount of active (free) ACC that might be used for ethylene production. We measured free and malonylated ACC at 1, 3, and 7 dai. Because previous studies showed that *P. indica* preferentially colonizes the maturation zone of roots [24], [25], the upper two centimeters of the root (basal part = maturation zone) were analyzed separately from the remaining apical root tissue (apical part). The amount of malonylated ACC was higher than free ACC indicative of a saturated ACC pool (Figure 2A, B). Significantly elevated amounts of free ACC were found in the apical root part during biotrophic colonization (3 dai) and in the apical as well as basal part during the cell-death associated growth phase (7 dai) (Figure 2A).

### MAMP-triggered root oxidative burst is suppressed by *P. indica*

**Figure 3:**
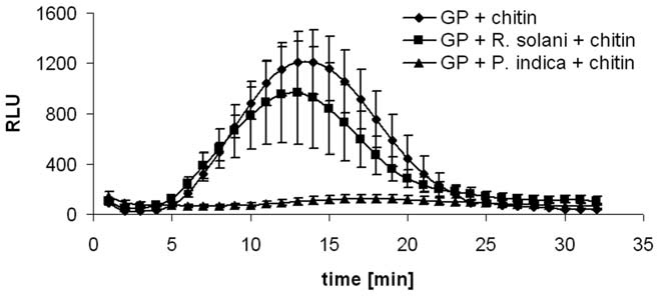
Suppression of chitin-induced oxidative burst by *P. indica*. Chitin (1 µM *N*-acetylchitooctaose) was applied to barley root segments of seedlings harvested at 3 days after *P. indica*- or *Rhizoctonia solani* inoculation or mock-treatment, respectively. Values are given as relative light units (RLU) over time as means with standard errors of two biological experiments with three independent measurements per treatment and experiment. GP, barley cv. Golden Promise.

Global gene expression analysis demonstrated that *P. indica* hardly induces defense responses in barley roots [27]. Consistent with this we showed that *P. indica* suppresses MAMP-triggered responses such as the oxidative burst and defense gene expression in *Arabidopsis* roots [24]. Ethylene signaling is required for MAMP-triggered oxidative burst, one of the earliest innate immune responses [13], [16]. Hence, the finding that *P. indica* induces ethylene synthesis genes (Table 1) and ACC synthesis during early (3 dai) and late colonization (7 dai) stages prompted us to assess the fungus' ability to suppress chitin-induced oxidative burst in barley roots. To this end, we determined chitin-induced root oxidative burst in non-colonized and *P. indica*-colonized roots. In non-colonized roots, a strong accumulation of H2O2 was measured after treatment with the octamer of fungal chitin (1 µM *N*-acetylchitooctaose, Figure 3) as was reported for leaves. In contrast, chitin-induced root oxidative burst was almost completely abolished in *P. indica*-colonized roots. This finding corroborates earlier results showing that *P. indica* has a strong capability in suppressing plant defense responses [24], [27]. Apparently, the anticipated increase in ethylene production does not result in a colonization-associated MAMP-triggered oxidative burst, or related signaling processes are suppressed by the fungus. To exclude the possibility that the suppressing activity is a general attribute of root colonizing fungi, we also tested the ROS-suppressing activity of *Rhizoctonia solani*, a pathogenic root-colonizing basidiomycete that also displays a broad host range. We found that *R. solani* could not suppress the chitin-induced ROS accumulation, suggesting that ROS-suppressing activity is associated with the symbiotic potential of *P. indica*.

### Colonization-associated induction of ACC synthases in *Arabidopsis* roots

**Figure 4:**
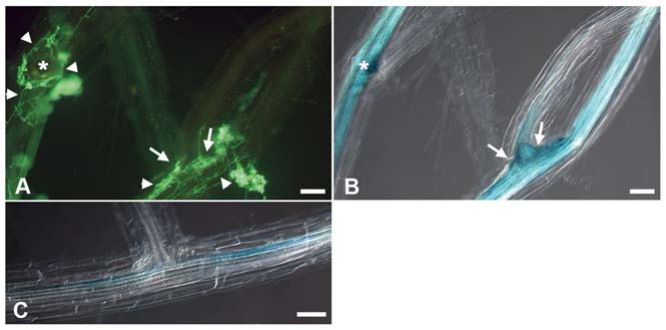
GUS accumulation in roots of *ACS1*::*GUS* reporter plants colonized by *P. indica*. *Arabidopsis* line *ACS1*::*GUS* was harvested at 7 dai and, after GUS and WGA-AF 488 staining, analyzed cytologically. (**A**, **B**) *P. indica* colonization at the base of lateral roots (arrows) or primordia (asterisks) of line *ACS1*::*GUS* was associated with enhanced GUS accumulation. *P. indica* (arrowsheads in A) was visualized by staining with WGA-AF 488. (**C**) In mock-treated *ACS1*::*GUS*, GUS staining was weakly detectable e.g. at the lateral root base. Bars = 60 µm.

**Figure 5:**
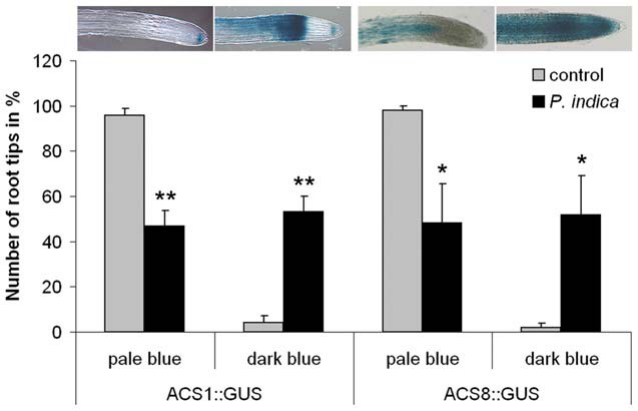
GUS accumulation in roots of *ACS1*::*GUS* and *ACS8*::*GUS* reporter plants colonized by *P. indica*. *Arabidopsis* lines *ACS1*::*GUS* and *ACS8*::*GUS* were harvested at 7 dai and, after GUS and WGA-AF 488 staining, analyzed cytologically. GUS staining was more pronounced in root tip regions of colonized roots as compared to mock-treated roots (upper images). At 7 dai, *P. indica*-colonized roots of both lines showed a significant increase of dark blue tips and a significant reduction in pale blue tips as compared to mock-treated roots. GUS staining did not colocalize with colonization sites of *P. indica* or extracellular fungal growth. The data base on at least two biological experiments. Asterisks indicate significant differences between control and *P. indica*-colonized roots according to Students *t*-test (* P<0.05, ** P<0.001).

ACC quantification in barley roots did not allow for cellular resolution of ACC production, nor did it prove an association of ACC synthesis with *P. indica* colonization. Since reporter lines for ACC synthesis are not available for barley, we took advantage of the *Arabidopsis*-*P. indica* system. We used *Arabidopsis*-reporter plants for ACC synthesis that express β-glucuronidase (GUS) fusions with promoters of genes encoding *1-aminocyclopropane-1-carboxylic acid synthases* (*ACS*). ACS are the rate limiting enzymes in ethylene synthesis [29]. In *Arabidopsis*, nine *ACS* genes (*ACS1*, *ACS2*, *ACS4*, *ACS5*, *ACS6*, *ACS7*, *ACS8*, *ACS9*, and *ACS11*) have been identified [29]. The respective reporter lines allowed monitoring of the spatio-temporal expression of an individual *ACS* gene upon *P. indica* colonization. To this end, *Arabidopsis* (reporter) plants were analyzed by fluorescence and bright field microscopy at 3 and 7 dai upon double-staining for GUS activity and for fungal hyphae with WGA-AF 488. Based on the AREX database [30], [31], all nine *ACS* genes are expressed in the meristematic, elongation, and maturation zone, but differ in level and site of expression level as well as site (Table S1). Among all the tested lines, only *ACS1*::*GUS* and *ACS8*::*GUS* showed a response to *P. indica* (Figure 4, 5). *ACS1* was induced by the fungus at primordia and the base of lateral roots at 7 dai (Figure 4). Most obviously and consistent with the ACC accumulation pattern in barley (see Figure 2), both *ACS1*::*GUS* and *ACS8*::*GUS* plants showed a strong GUS activation at 7 dai at the root tip region of *P. indica*-colonized plants (Figure 5) although the staining pattern slightly differed among both lines. GUS activity in *ACS1*::*GUS* was detected in the elongation zone, while GUS accumulated also in the meristem of ACS8::*GUS* (Figure 5). However, the GUS accumulation pattern did not differ in any line in non-colonized compared to *P. indica*-colonized roots at 3 dai.

### Ethylene signaling enhances colonization of *Arabidopsis* roots by *P. indica* at the cell death-associated interaction stage

**Figure 6:**
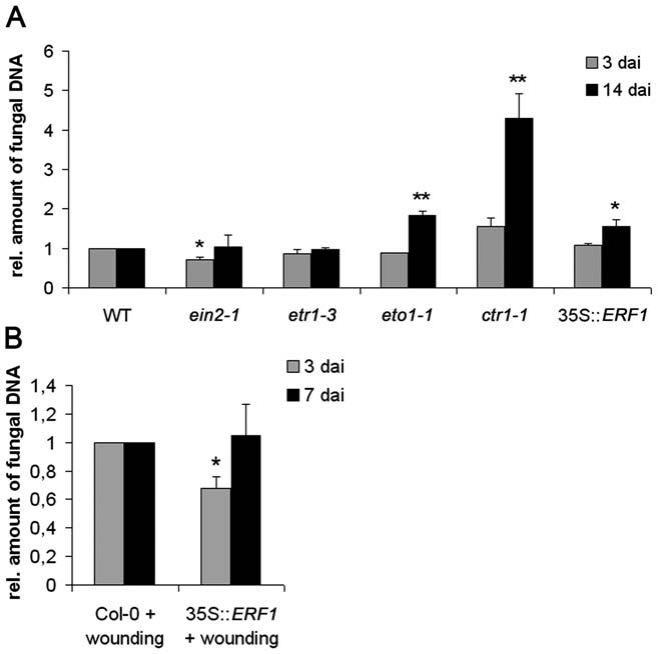
Colonization of ethylene synthesis and signaling mutants by *P. indica*. (**A**) Three-week-old plants were inoculated with *P. indica* and fungal biomass was determined in *ein2-1*, *etr1-3, eto1-1, ctr1-1*, and *35S*::*ERF1* by qRT-PCR at 3 and 14 dai. (**B**) Three-week-old *35S::ERF1* plants were injured with foreceps and inoculated with *P. indica* at 1 day after wounding. Fungal biomass was determined by qRT-PCR at 3 and 7 dai. All values were related to Col-0 (set to one). The data are based on at least three independent experiments. Students *t*-test indicated significant difference in *P. indica*-colonization (* P<0.05, ** P<0.001).

To further confirm in *Arabidopsis* that ethylene affects *P. indica* colonization, we quantified fungal growth in the *Arabidopsis* mutants *etr1-3* and *ein2-1*, which are impaired in ethylene signaling, as well as *ctr1-1*, which shows constitutive ethylene signaling. In addition, the ethylene synthesis mutant *eto1-1* was tested. Quantitative real time (qRT)-PCR-based quantification of the amount of fungal DNA at 3 dai (biotrophic colonization) and 14 dai (cell death-dependent colonization) showed higher colonization of mutants that displayed constitutive ethylene signaling (*ctr1-1*) or enhanced ethylene synthesis (*eto1-1*) during cell death-associated colonization. Comparable to MCP treatment of *Arabidopsis* roots (Figure 1B), colonization of *ein2-1* was reduced at 3 dai and reached wild type levels at 14 dai. Colonization of *etr1-3* was not altered as compared to the wild-type (Figure 6A). Subsequently, we analyzed the colonization of 35S::*ERF1* plants. ERF1 is a transcription factor that is central to ethylene-associated defense signaling in *Arabidopsis* [11]. Like *ctr1-1* and *eto1-1*, plants overexpressing *ERF1* were significantly more colonized by *P. indica* at 14 dai (Figure 6A). Improved colonization of 35S::*ERF1* is contradictory to a recent study, which demonstrated unaltered colonization of this line at 12 dai [32]. Most probably, the divergent experimental set up resulted in the different outcomes. In our assay, plants were grown on sugar-free medium in square petri dishes to which a defined amount of spores (500,000 spores ml^−1^) was directly applied to roots, thereby avoiding detachment of seedlings and roots (see Materials and Methods). Detachment of roots might result in root injuries and activation of wound-induced stress signaling which might affect root colonization. Consistent with this, when we slightly injured roots with forceps and inoculated these roots one day later, we observed reduced colonization of 35S::*ERF1* roots at 3 dai, while colonization was unaltered at 7 dai (Figure 6B).

## Discussion

The spatio-temporal events associated with the colonization of barley and *Arabidopsis* roots by *P. indica* are very similar, including a biotrophic followed by a cell death-dependent colonization phase [24], [25], [26], [27], [33]. Our analyses suggest fungus-induced ethylene production especially in apical root parts of barley and *Arabidopsis* since we detected enhanced ACC production in barley (Figure 2) and induction of *ACS1* and *ACS8* in *Arabidopsis* (Figure 5). *ACS1* induction has not been reported in roots [29], but is in accordance with the AREX database prediction (Table S1). The analyses suggest systemic regulation of *ACS1* and *ACS8* as the fungus was not detected at root apices. Notably, ACC is a mobile molecule and not necessarily produced at sites of ethylene action. ACC produced in roots is known to be transported via the xylem to allow ethylene synthesis in distant tissue [34], [35], [36]. Ethylene, like jasmonic acid (JA) and salicylic acid (SA), effectively sustains MAMP-triggered immune responses against pathogens [2], [37], [38], and also affects mutualistic symbioses, since ethylene inhibits mycorrhization and rhizobial nodulation of legumes [39], [40], [41], [42]. However, *P. indica*-induced ACC production is probably not participating in early immune signaling (e.g. oxidative burst). First, based on leaf expression data in Genevestigator [43], *ACS1* and *ACS8* are not responsive to biotic stress. Secondly, as already reported for *Arabidopsis* roots [24], *P. indica* also suppressed MAMP-induced oxidative burst in barley roots (Figure 3). Thirdly, ethylene significantly supported *P. indica* colonization in both plants (Figure 1, 6). Blockage of ethylene signaling by MCP (Figure 1B) or by the lack of EIN2 resulted in reduced root colonization at 3 dai in *Arabidopsis* (Figure 6A), while MCP treatment caused reduced colonization of barley at 7 dai (Figure 1A). The temporally different effect of ethylene signaling on compatibility (Figure 1) suggests differences in fungal requirements to colonize both plants and indicates *P. indica*'s adaptive potential to colonize root cells of different species. It will be interesting to see in future studies which ethylene-regulated processes are influenced by the fungus in both plants. Fourthly, *ctr1-1*, *eto1-1* and *35S*::*ERF1* plants that display constitutive ethylene signaling, synthesis or defense, respectively, were significantly better colonized by *P. indica* at 14 dai (Figure 6). The stunted root morphology of *eto1-1* and *ctr1-1* may contribute, but cannot entirely account for improved colonization, as we also observed increased colonization in *35S*::*ERF1* plants, which possess an unaltered root phenotype. Notably, lack of *etr1* did not affect root colonization as seen in *ein2-1* mutants (Figure 6) and suggests redundancy among the five ethylene receptors during *P. indica* colonization of *Arabidopsis* roots, which is not observed for the downstream effector EIN2. Consistent with this, blockage of ethylene perception by MCP resulted in reduced colonization at 3 dai as observed for *ein2-1* (Figure 1, 6).

Interestingly, the pronounced GUS accumulation in *P. indica*-colonized *ACS8*::*GUS* roots (Figure 5) is reminiscent of its induction in *Arabidopsis* roots after auxin treatment [29]. Notably, auxin stimulates the activities of several ACS [44], [45] and antagonizes SA-mediated defense [46]. In turn, SA defense restricts *P. indica* colonization, while JA signaling, in analogy to ethylene signaling, supported root colonization [24]. An antagonistic activity of ethylene to SA-related defense has been demonstrated in 35S::*ERF1* seedlings [47], which displayed enhanced susceptibility to *P. indica* (Figure 6). It is tempting to speculate that auxin might be synthesized by the plant [27], and/or by the fungus as was recently hypothesized [48]. It would be interesting to investigate in future, whether auxin metabolism might be activated during *P. indica* colonization, thereby regulating *ACS8* (and *ACS1*) expression and impairing SA-related immune processes.

The inconsistency of our results on the colonization of 35S::*ERF1* (Figure 6A, B) in comparison to a recent report [32] is most probably explained by the different colonization assays used in the two studies. In addition, we determined fungal colonization in a direct approach by quantifying fungal DNA in relation to plant DNA via qRT-PCR. We found this to be essential for a sensitive quantification of fungal DNA in roots.

Taken together, we demonstrated that ethylene supports colonization of barley and *Arabidopsis* roots by *P. indica*. This is in line with the requirement of JA for *Arabidopsis* root colonization by *P. indica* [22], considering the synergistic activity of JA and ethylene in plant signaling [44]. Recent studies revealed the significance of ethylene in JA-SA crosstalk. While SA blocked JA signaling, this inhibition did not occur in plants in which JA and ethylene pathways were activated [49]. It is tempting to speculate that *P. indica* recruits ethylene together with JA in order to outcompete SA immunity. Recent studies demonstrated the effectiveness of SA-related immunity to restrict root colonization by *P. indica* [22]. Interestingly, JA and ethylene are required for induced systemic resistance (ISR) observed in leaves after colonization of roots by beneficial microbes such as *Pseudomonas fluorescens* [50], [51]. Therefore, recruitment of JA/ethylene during root colonization might be further connected to systemic resistance that is induced in leaves of *P. indica*-colonized *Arabidopsis* [52]. Indeed, systemic resistance induced by *P. indica* was dependent on JA signaling and suggested to be based on ISR [52].

## Materials and Methods

### Plant material and fungal inoculation

Seeds of *Arabidopsis thaliana* ecotype Col-0 and mutants *eto1-1* (N3072), *ctr1-1* (N8057), *etr1-3* (N3070), *35S*::*ERF1* (N6142), and *ACS*::*GUS* reporter plants (N31379, N31380, N31381, N31382, N31383, N31385, N31386, N31387) were obtained from the European Arabidopsis Stock Centre (NASC). All the *Arabidopsis* plants and the respective parents were grown on ½ Murashige and Skoog medium on square petri dishes, which were vertically positioned. Plants were grown at 22/18°C day/night cycle under short-day conditions (10 hours light) at 60% relative humidity in a growth chamber. Three-week-old plants were inoculated with 500,000 *P. indica* chlamydospores ml^−1^ (DSM11827 from Deutsche Sammlung von Mikroorganismen und Zellkulturen, Braunschweig, Germany) by adding 1 ml spore suspension per petri dish containing ∼ 40 plants and harvested at indicated timepoints. For root injury experiments, 35S::*ERF1* plants were grown for three weeks on ½ Murashige and Skoog medium on squared petri dishes and thereafter scratched with a foreceps. One day later roots were inoculated with *P. indica* and harvested at 3 and 7 dai. For barley, all experiments were conducted with cultivar Golden Promise. Golden Promise is a barley line that is no longer commercially available and the seeds used for our studies derive from our own annual propagation. Plants were inoculated as described previously [25]. In brief, barley kernels were sterilized with 6% sodium hypochloride, rinsed in water, and germinated for 2 days. Subsequently, seedling roots were immersed in an aqueous solution of 0.05% Tween-20 containing 500,000 spores ml^−1^ of *P. indica* chlamydospores. Inoculated seedlings were transferred to 1.5 L glass jars containing plant nutrient medium (PNM)(1/10) [27]. Barley root treatment and harvest was performed as described below.

### Cyto-histological observations and β-glucuronidase (GUS)-based studies

For cytological examinations, the fungus was stained with wheat germ agglutinin-Alexa Fluor 488 (WGA-AF 488) as previously described [25]. *Arabidopsis ACS*::*GUS* plants were harvested at indicated timepoints. GUS staining was performed as described previously [53]. Briefly, roots were stained with staining solution (50 mM phosphate buffer, pH 7.0, 0.5 mM potassium ferricyanide, 0.2% Triton X-100, 0.5% DMSO, 20% methanol, 2 mM EDTA, 1 mM X-Gluc) and incubated overnight at 37°C. The staining reaction was stopped by incubation in 70% ethanol. The roots were analyzed microscopically using an Axioplan 2 microscope (Carl Zeiss, Jena, Germany). WGA-AF 488 was detected at 470/20 nm (excitation) and 505–530 nm (emission).

### Application of ACC and 1-methylcyclopropene (MCP)

Two-day-old barley seedlings (cv. Golden Promise) were inoculated with *P. indica* and transferred to PNM_(1/10)_ [27] supplemented with 100 µM ACC (Sigma-Aldrich, Munich, Germany). ACC was dissolved in water and filter-sterilized prior to its addition to autoclaved plant growth media. For MCP (Rohm and Haas Company, Philadelphia, USA) treatment, inoculated barley seedlings were transferred to glass jars (volume 1.5 l) in which a vial was placed containing 16 mg MCP (0.14% active ingredient) dissolved in 200 µl water. For *Arabidopsis*, vials containing MCP were placed inside petri dishes in which plants were grown and inoculated as described above. MCP treatment was conducted immediately after inoculation of roots with *P. indica*. The final concentration of 1-MCP in the gas phase of the jar and petri dishes was expected to be about 500 ppt [54]. Roots were harvested at 3 and 7 dai, frozen in liquid nitrogen and subjected to DNA isolation (see below).

### Determination of ACC content

Two-day-old barley plants (cv. Golden Promise) were inoculated with *P. indica* or mock treated and transferred to jars containing PNM_(1/10)_ [27]. Roots were harvested at 0, 1, 3, and 7 dai. At 3 and 7 dai, the upper two centimeters (basal part) were harvested separately from the lower apical part. Plant material was ground in liquid nitrogen and extracted according to Langebartels et al. (1991) [55]. Free ACC and total ACC released by acid hydrolysis (2 N HCl for 3 h at 120°C) were determined [55], [56]. The amount of conjugated ACC was calculated by subtracting the amount of ACC from total ACC.

### Quantitation of *P. indica* colonization by qRT-PCR

Genomic DNA of wild type and *Arabidopsis* mutant roots as well as ACC-/MCP- and mock-treated barley roots was extracted from ∼100 mg root material using Plant DNeasy Kit (Qiagen, Hilden, Germany) according to the manufacturer's instructions. Ten ng of total DNA served as template for qRT-PCR analyses. Amplifications were performed in 20 µl SYBR green JumpStart Taq ReadyMix (Sigma-Aldrich, Munich, Germany) with 350 nM oligonucleotides, using an Mx3000P thermal cycler with a standard amplification protocol (Stratagene, La Jolla, USA). Fungal colonization was determined by the 2^−ΔCt^ method [57] by subtracting the raw threshold cycle (Ct) values of *P. indica Internal Transcribed Spacer* (*PiITS*) gene from those of *AtUBQ5* (At3g62250) or *HvUBQ* (NIASHv1058N10), respectively, using the *PiITS*-specific primers 5′-CAACACATGTGCACGTCGAT-3′ and 5′-CCAATGTGCATTCAGAACGA-3′ (slope: −3.208, Y-intercept: 30.55, R^2^: 0.995, efficiency [%]: 104.966), *AtUBQ5*-specific primers 5′-CCAAGCCGAAGAAGATCAAG-3′ and 5′-ACTCCTTCCTCAAACGCTGA-3′ (slope: -3.281, Y-intercept: 27.938, R^2^: 0.998, efficiency [%]: 101.754), or *HvUBQ*-specific primers 5′-ACCCTCGCCGACTACAACAT-3′ and 5′-CAGTAGTGGCGGTCGAAGTG-3′ (slope: −3.212, Y-intercept: 24.559, R^2^: 0.988, efficiency [%]: 104.783).

### Chitin-induced root oxidative burst

Three-day-old barley seedlings were either treated with *P. indica*, *Rhizoctonia solani* AG8, or mock-treated. For determination of oxidative burst, roots were cut in 1 cm long pieces (10 mg per assay) at 3 dai and floated in water over night. Roots were transferred to tubes with 20 µM luminol (Sigma-Aldrich, Munich, Germany) and 1.5 µg horseradish peroxidase (Roche Diagnostics, Mannheim, Germany). One µM *N*-acetylchitooctaose was used as elicitor for a luminol-based assay [58]. Luminescence measurements were performed for 30 min in a Berthold Lumat LB 9501 (Berthold, Bad Wildbach, Gemany).

### Statistical analysis

All experiments were conducted at least in duplicate and standard errors were calculated for all mean values. Levels of significance were calculated using Student's *t*-test.

## Supporting Information

Table S1 **Regulation of** ***ACC synthase*** **genes in** ***Arabidopsis*** **roots according to the AREX database.**
(DOC)
