# Supplementary material for: Ethylene Supports Colonization of Plant Roots by the Mutualistic Fungus Piriformospora indica
Source: PLoS One. 2012 Apr 19;7(4):e35502. doi: 10.1371/journal.pone.0035502 (PMC3334895; doi:10.1371/journal.pone.0035502)
Supplement: Table S1 — Regulation of ACC synthase genes in Arabidopsis roots according to the AREX database. (DOC) [file pone.0035502.s001.doc]

**Supporting Information**

**Table S1.** Regulation of *ACC synthase* genes in *Arabidopsis* roots according to the AREX database.

|  | ACS1 | ACS2 | ACS4 | ACS5 | ACS6 | ACS7 | ACS8 | ACS9 | ACS11 |
| --- | --- | --- | --- | --- | --- | --- | --- | --- | --- |
|  | AT3G61510 | AT1G01480 | AT2G22810 | AT5G65800 | AT4G11280 | AT4G26200 | AT4G37770 | AT3G49700 | AT4G08040 |
| Lateral Root Primordia |  | + | + |  | ++ | + | ++ |  | + |
| Phloem CCs |  | + | + |  | ++ |  | + |  |  |
| Meta/Proto Phloem |  | + | + | + | ++ |  | ++ | + | + |
| Meta/Proto Xylem |  | + | + |  | ++ |  | ++ |  |  |
| Phloem Pole Pericycle |  | ++ | + |  | ++ | + | ++ |  | + |
| Xylem Pole Pericycle |  | + | + |  | ++ |  | ++ |  | + |
| Procambium | ++ |  | ++ | ++ |  |  | + | ++ | + |
| Endodermis | + | ++ | + | + | ++ | + | ++ | + | + |
| Cortex |  | + | + |  | ++ | + | + | + | + |
| Non-Hair | + | + | ++ | + | ++ | + | + | + | + |
| Hair |  | + | ++ | + | ++ | + | + | + | + |
| Quiescent Center |  | ++ | + |  | + |  | + |  |  |
| Lateral Root Cap | + | + | + | + | + | + | + | + | + |
| Columella |  | + | + |  | ++ |  | + |  |  |
|  | | | | | | | | | |
| Meristmatic Zone | + | + | + | + | + | + | + | + | + |
| Elongation Zone | + | + | + | + | ++ | + | + | + | + |
| Maturation Zone | + | + | ++ | + | ++ | ++a | +b | + | +c |
|  |  |  |  |  |  |  |  |  |  |
| + Min. Exp. (≥ 0.5) |  |  |  |  |  |  |  |  |  |
| ++ Max. Exp. (≥ 2) |  |  |  |  |  |  |  |  |  |
| a predominant expression in maturation zone 9-11 | | | |  |  |  |  |  |  |
| c predominant expression in maturation zone 12 | | | |  |  |  |  |  |  |
| b predominant expression in maturation zone 11 | | | |  |  |  |  |  |  |
